# Observation of Zn Dendrite Growth via Operando Digital Microscopy and Time-Lapse Tomography

**DOI:** 10.1021/acsami.2c19895

**Published:** 2023-03-09

**Authors:** Wenjia Du, Zhenyu Zhang, Francesco Iacoviello, Shangwei Zhou, Rhodri E. Owen, Rhodri Jervis, Dan J. L. Brett, Paul R. Shearing

**Affiliations:** † Electrochemical Innovation Lab, Department of Chemical Engineering, University College London, London WC1E 7JE, U.K.; ‡ The Faraday Institution, Quad One, Harwell Science and Innovation Campus, Didcot OX11 0RA, U.K.

**Keywords:** zinc electrodeposition, dendrites, plating, stripping, X-ray computed tomography

## Abstract

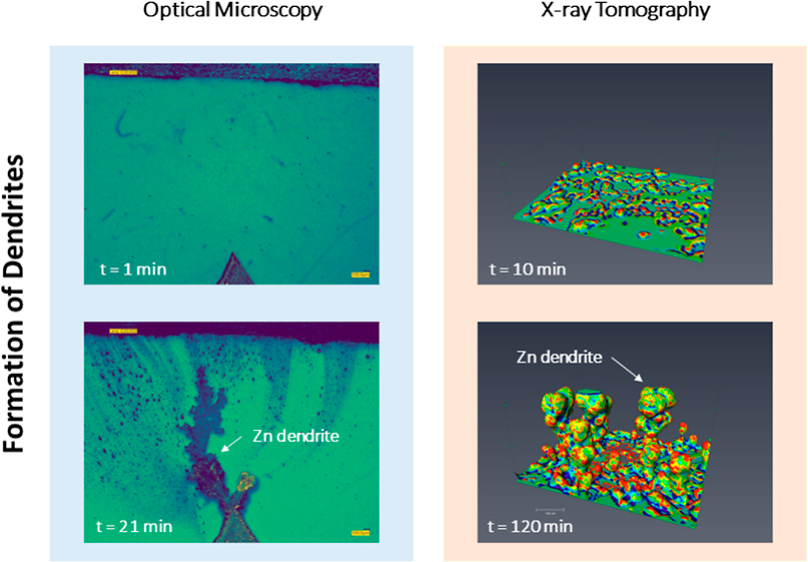

The zinc-ion battery is one of the promising candidates for next-generation energy storage devices beyond lithium technology due to the earth’s abundance of Zn materials and their high volumetric energy density (5855 mA h cm^–3^). To date, the formation of Zn dendrites during charge–discharge cycling still hinders the practical application of zinc-ion batteries. It is, therefore, crucial to understand the formation mechanism of the zinc dendritic structure before effectively suppressing its growth. Here, the application of operando digital optical microscopy and in situ lab-based X-ray computed tomography (X-ray CT) is demonstrated to probe and quantify the morphologies of zinc electrodeposition/dissolution under multiple galvanostatic plating/stripping conditions in symmetric Zn||Zn cells. With the combined microscopy approaches, we directly observed the dynamic nucleation and subsequent growth of Zn deposits, the heterogeneous transportation of charged clusters/particles, and the evolution of ‘dead’ Zn particles via partial dissolution. Zn electrodeposition at the early stage is mainly attributed to activation, while the subsequent dendrite growth is driven by diffusion. The high current not only facilitates the formation of sharp dendrites with a larger mean curvature at their tips but also leads to dendritic tip splitting and the creation of a hyper-branching morphology. This approach offers a direct opportunity to characterize dendrite formation in batteries with a metal anode in the laboratory.

## 1 Introduction

In pursuit of higher energy and power densities for electronic devices and electric vehicles, various types of electrochemical energy storage devices have been successfully developed in recent decades. Commercial rechargeable ion batteries [(such as the lithium-ion battery (LIB)] are typically based on the intercalation or electrochemical alloying of ions in positive and negative electrode materials, which to date have played the most significant role in the market.^1^ However, it remains challenging to fulfill the fast-growing demands for large-scale energy storage due to the challenges of conventional LIB electrode materials, such as their limited specific capacity and volume expansion.^2^ Also, LiBs heavily rely on the supply of critical materials, such as Li, Co, and Ni. These prominent battery-based minerals will face global supply chain risks in the near future due to their high demand, geopolitical events, and logistical disruption.^3^ By 2040, it is expected that 4 times as many critical minerals as it does today would be needed for renewable energy technologies.^4^ Hence, rechargeable multivalent batteries with metal anodes (such as Zn, Ca, Mg, Al, etc.) have been considered promising candidates for next-generation batteries due to their high theoretical energy density, low redox potential, low cost, and improved safety.^5,6^ However, the common challenge for the commercialization of these batteries with metal anodes is the poor cycling performance and safety issues caused by dendrite growth.^7−10^

For the Zn metal battery with a Zn metal anode and a Zn^2+^ ion storage cathode, normally, metal oxides such as MnO_2_ is an attractive choice because of its ultralow cost, ultrahigh volumetric energy density, and compatibility in safe aqueous electrolytes.^11^ Unfortunately, the Zn metal anode suffers from dendrite formation and hydrogen generation during the plating/stripping of Zn ions.^12^ Additionally, side reactions (for example, electrode corrosion in mild electrolytes and passivation in alkaline electrolytes^13^) that consume the electrolyte and Zn metal result in “dead zinc”, low Coulombic efficiency, and short-circuiting of the cells.^14^ A series of strategies for suppressing dendrite growth and side reactions of Zn metal anodes have been proposed in recent years, including structural design of the electrode,^15^ modification of the electrode–electrolyte interface,^16^ and optimization of the electrolyte composition.^17^

During the electroplating of Zn, Zn^2+^ ion diffusion, reduction, nucleation, and crystal growth occur in succession. Zn nucleation is a diffusion-controlled process, which is affected by the electric field and ion distribution.^18^ Due to the inhomogeneous nucleation and preferential accumulation, Zn dendrites continuously grow at the large curvature radius tip, where the localized electric field is higher.^19^ After repeated uneven plating-stripping, Zn dendrites could cause short-circuiting of the cell by piercing separators. The factors that influence the growth of Zn dendrites include, but are not limited to, the electrode structure and material, local ion concentration gradient and mobility, electrolyte composition, and temperature. In particular, electrochemical operation conditions such as the current density and plating-stripping capacity also heavily influence the structure of the formed Zn dendrites.^20^

In situ characterization of Zn dendrite growth is crucial to understand the mechanisms of dendrite formation and thus to design reliable electrodes for rechargeable metal batteries with improved plating-stripping performance. Recent works have shown the initial structure of Zn crystals by in situ atomic force microscopy (AFM),^21^ the nanoscale or microscale structures of Zn dendrites by ex-situ electron microscopy, the lateral morphology evolution of the Zn anode surface by in situ optical microscopy,^22^ and dendrite formation, dissolution, and regrowth by operando synchrotron X-ray tomography.^23,24^ However, many of these methods lack real-time information, and the destructive approach or requirement of bespoke cells may not represent genuine working environments. Although synchrotron X-ray imaging provides an opportunity for high-throughput operando studies, this technique is difficult to access, and some compromises are required in the cell geometry.

In this study, we have developed a facile configuration for operando probing of Zn dendritic formation in Zn||Zn symmetric cells by galvanostatic electrodeposition. We also present a novel combination of operando digital optical microscopy and in situ lab-based X-ray CT. Using both advanced characterizations, the correlations between the evolution of the microstructure and the electrochemical condition (i.e., current density) are revealed. This work provides an in-depth study of the nucleation and growth mechanisms of Zn dendrites, and such correlative approaches can be applied to other metal anode batteries.

## 2 Experimental Methods

### 2.1 Materials, Battery Assembly, and Electrodeposition

All cells were assembled in an ambient environment. 3 M ZnSO_4_ (ZnSO_4_·7H_2_O, ACS reagent, 99%, Sigma-Aldrich) in deionized water was used as an electrolyte. Thin zinc foil (purity: 99%, thickness: 0.5 mm, Sigma-Aldrich) was manually trimmed as the electrodes of a symmetric Zn||Zn cell (a Zn triangle substrate and a Zn foil), which is accommodated by two planar quartz plates (5 × 5 mm) in ambient atmosphere. The cells were charge/discharge cycled at three different currents (1, 6, and ±10 mA, corresponding to the current densities of ca. 0.03, 0.18, and ±0.3 mA cm^–2^) for high-throughput, operando, optical microscopy. It should be noted that a “triangle-like” substrate was used for all operando microscopicelectrodeposition studies. Therefore, the top Zn electrode was used to calculate the current density since this value is not constant for the triangle electrode. The associated electrochemical profiles can be found in Figure S1. No separator was used, and a distance of ca. 3.5 mm between two Zn electrodes was maintained in this setup. For 4D X-ray imaging, Swagelok cells were assembled with two laser-machined Zn disc electrodes (diameter of ca. 2.6 mm) and a glass fiber (Whatman glass microfiber filters, grade GF/D, diameter of 2.9 mm) separator soaked in the same electrolyte. Two different currents (0.1 and 1 mA, corresponding to the current densities of 2 and 20 mA cm^–2^) were applied to the Zn||separator||Zn cell for 4D imaging of dendritic growth. A GAMRY 1000E potentiostat (GAMRY Instruments, USA) equipped with Gamry Framework software was used to control the electrodeposition process. To correlate the imaging information to cell performance, two long-term electrochemical measurements of the Swagelok cells were also performed under identical conditions.

### 2.2 Operando Digital Microscopy and Batch Image Processing

Operando observation of dynamic dendritic formation and dissolution within the symmetric Zn||Zn cell was performed with a 4 K digital microscope (Keyence VHX-7000, Japan). The experimental setup is shown in Figure S2. A 50× objective lens was used to obtain a resolution of ca. 0.5 μm, resulting in a relatively large field of view (FOV) of 5.8 × 4.2 mm^2^. The image acquisition was synchronized with electrochemical measurements. During low and medium constant current density modes (0.03 and 0.18 mA cm^–2^), the digital images were continuously taken until cell short-circuiting, with one frame per minute. In the high current density mode (0.3 mA cm^–2^), the video mode was switched on to enable faster image acquisition to capture the dynamic growth. Afterward, the raw RGB images were converted to 8 bit images in ImageJ.^25^ These datasets were batch-processed and tracked using an in-house algorithm implemented in Spyder (an open-source Python platform), including automated segmentation using the Otsu method.^26^ The area, volume, and height of Zn dendrites were also quantified. In Figure S3, the data processing procedure for quantification of “dead” Zn was performed in Avizo 2022.1 (ThermoFisher Scientific, USA). The raw 8 bit image was initially cropped before the binarization (dendrites and background). Then, the unconnected features were manually selected and classified as the “dead” Zn from all dendrites.

### 2.3 X-ray Microscopy and Image Processing

The Zn||separator||Zn Swagelok cell was scanned in the fresh state as well as after 10-, 30-, 60-, and 120 min electrodeposition under two different current densities (2 and 20 mA cm^–2^) using a Nikon XTH 225 instrument (Nikon Metrology, Tring, UK). Each scan was carried out using a tungsten target, a voltage of 100 kV, and a total power of 6 W. In total, 3185 projections were obtained with an exposure time of 1 s to maximize the image quality, resulting in each tomogram having a fast acquisition time of ca. 50 min. Both Zn electrodes were maintained within the FOV to obtain a spatial resolution of ca. 5.5 μm. The experimental setup is shown in Figure S4. The obtained projections were reconstructed using Nikon CT Pro 3D software (Version XT 4.4.4, Nikon Metrology, Tring, UK). A median filter was applied to these reconstructed datasets before the image segmentation. Both overall and local microstructures are rendered and presented in 3D. A sub-volume sequence was cropped to a size of 600 × 800 × 210 voxels in the middle of the associated entire volumes. 3D visualization and curvature analysis were performed in Avizo 2022.1.

## 3 Results and Discussion

Initially, the dynamic electrodeposition and dissolution of Zn in the aqueous electrolytes were characterized using high-throughput operando digital microscopy under three constant currents (CASE 1 to 3). Time-lapse X-ray microscopy was then used to probe the microstructural evolution of Zn dendrite growth in 4D (3D plus time) under two constant currents (CASE 4 and 5). Both experimental approaches have their merits and complement each other to contribute to understanding the Zn metal plating and stripping processes.

### 3.1 Dynamic Observation of Zn Electrodeposition and Dissolution

Operando visualization techniques (i.e., digital microscopy) reveal the topological evolution of deposited Zn. Such an experimental setup provides a low-cost, dynamic observation capability at the microscale. A recent study using a very similar setup at the nanoscale has been reported.^27^

**Figure 1:**
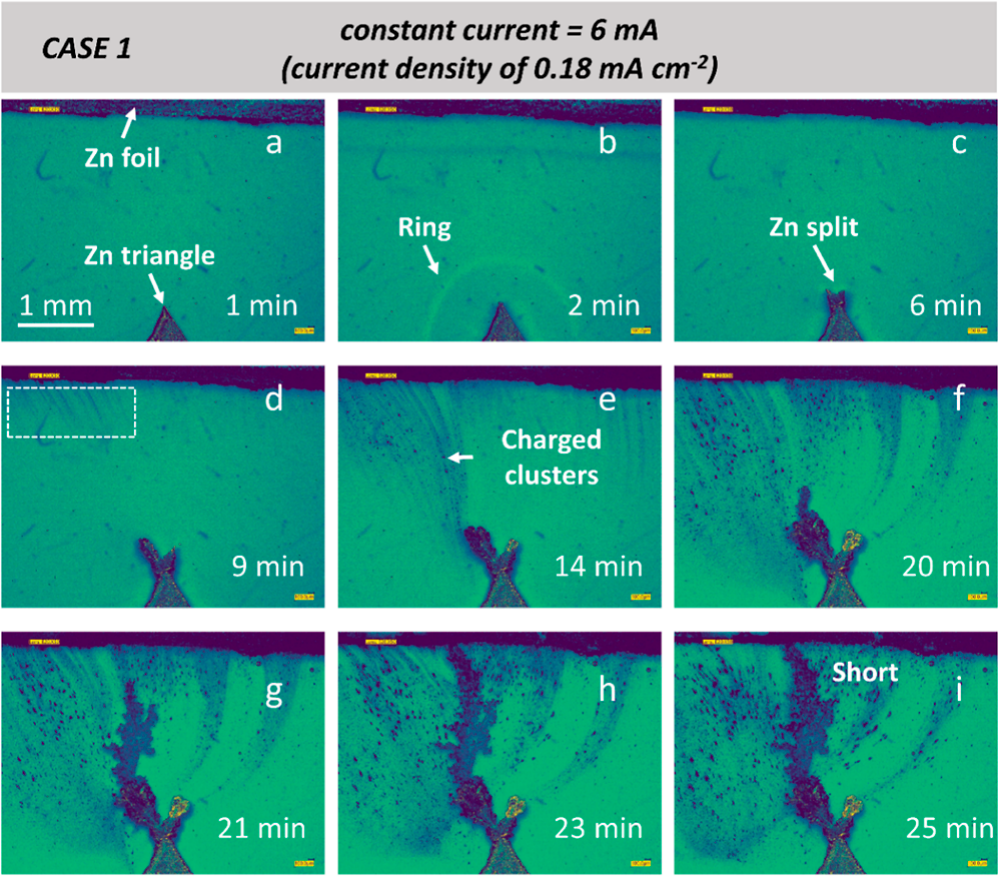
Operando imaging of the fractal zinc deposition under 6 mA (current density of 0.18 mA cm^–2^). The zinc dendritic growth in a symmetric zinc||zinc cell at a constant current of 6 mA until short-circuiting at room temperature. The morphological evolution of Zn electrodeposition after (a) 1, (b) 2, (c) 6, (d) 9, (e) 14, (f) 20, (g) 21, (h) 23, and (i) 25 min. It should be noted that the image sequence has been automatically segmented, labeled, and quantified (colors of dark blue and green represent the solid zinc and liquid electrolyte phases, respectively).

**Figure 2:**
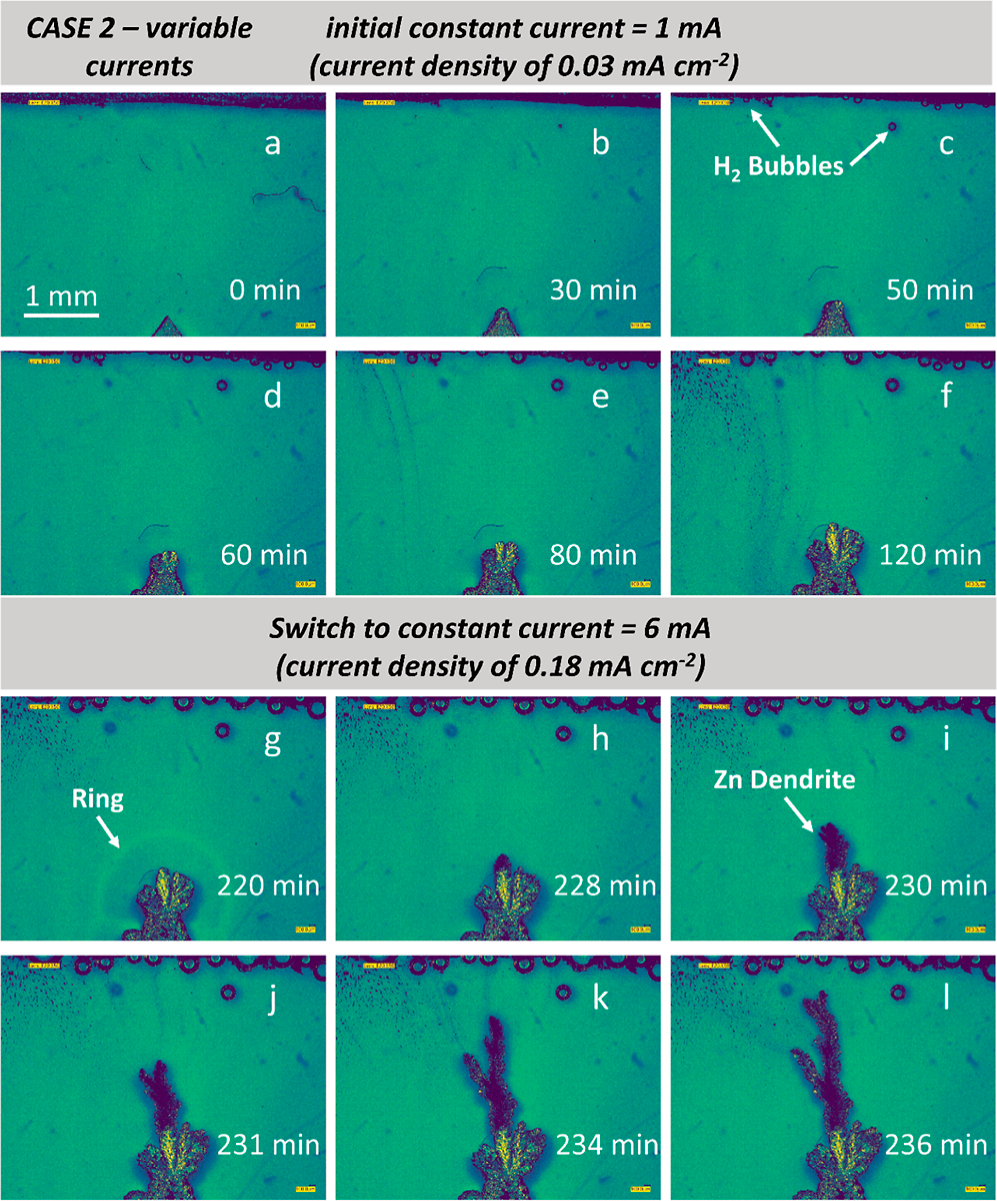
Operando imaging of zinc electrodepositions using variable currents increasing from 1 to 6 mA (current densities from 0.03 to 0.18 mA cm^–2^). The nucleation and crystallization of zinc dendrites in the same symmetric zinc||zinc cell at room temperature. The slower zinc electrodeposition at a low current of 1 mA for (a) 0, (b) 30, (c) 50, (d) 60, (e) 80, and (f) 120 min. Continuation of the faster zinc electrodeposition using a medium current of 6 mA for (g) 220, (h) 228, (i) 230, (j) 231, (k) 234, and (l) 236 min.

**Figure 3:**
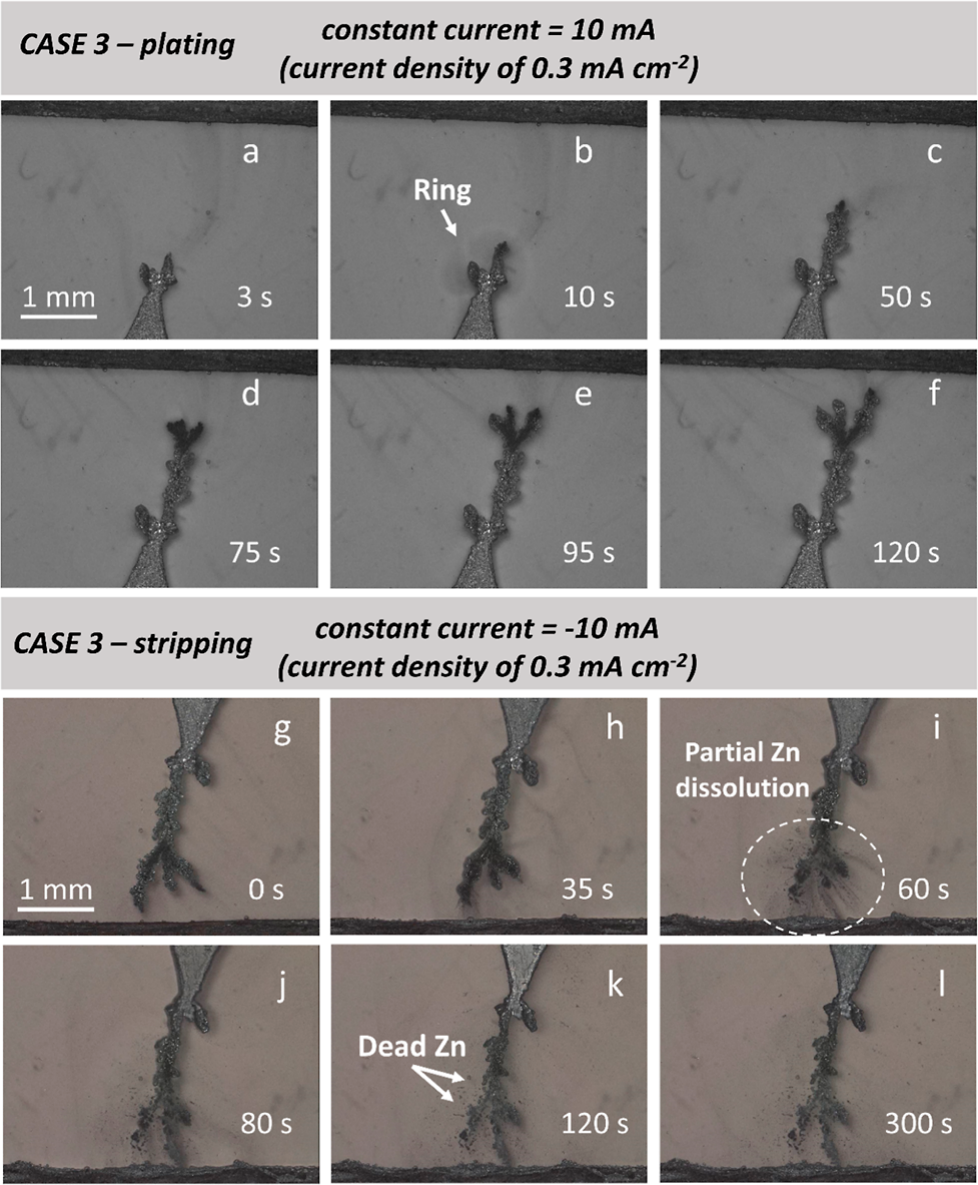
Formation and dissolution of Zinc dendrites under 10 and −10 mA (current densities of 0.3 and −0.3 mA cm^–2^). The evolution of zinc in a symmetrical zinc||zinc cell during plating and subsequent stripping. The fastest zinc electrodeposition in this study at a high current of 10 mA after (a) 3, (b) 10, (c) 50, (d) 75, (e) 95, and (f) 120 s. The partial dissolution of the same Zn dendrite and the formation of dead zinc particles at the local area by applying a negative current of −10 mA after (g) 0, (h) 35, (i) 60, (j) 80, (k) 120, and (l) 300 s. It should be noted that we did not label the image series because images are extracted from videos that are unsuitable for image segmentation (higher temporal resolution but lower spatial resolution).

Figure 1 and Movie 1 (all movies can be found or downloaded in the Supporting Information) illustrate the nucleation and growth of deposited Zn during plating under 0.18 mA cm^–2^. At *t* = 1 min, no obvious newly formed features are observed (Figure 1a), and the system is under the equilibrium condition according to the Nernst–Planck equation.^28^ However, a “ring-like” feature indicated by a white arrow in the electrolyte and a small Zn protrusion at *t* = 2 min are observed in Figure 1b. The phenomenon of the “ring” feature was also found in all other CASEs (see Figures 2 and 3). This ring may be regarded as a “boundary”, where the electrolyte concentration is different at the inner and outer of the ring. A “ring” feature appears in the electrolyte, signaling that the critical conditions for breaking equilibrium have been reached, which are driven by the divergent electric field.^29^ The irregular ion migration leads to Zn^2+^ ion aggregation and Zn nucleation. Figure 1c shows the Zn electrodeposit split into two dendritic tips at *t* = 6 min. The left tip seems slightly higher than the right one due to the uneven ion distribution. After *t* = 9 min, in Figure 1d, more intensive, irregular ion migration indicates the uneven mass transportation is occurring at the top-left corner, as highlighted in the white dashed square. The left tip grew faster and had a larger branch size than the right one, due to higher ion aggregation. The phenomenon of uneven Zn^2+^ motion will lead to further dendrite branching and irregular mass convection. Figure 1e,f demonstrates the formation of secondary and tertiary dendrites on the left branch at *t* = 15 and *t* = 20 min, respectively. In Figure 1e–i, numerous particles (colored dark blue) with a size up to ca. 100 μm can be seen moving from the top to the bottom side. These particles are suspected to be the charged clusters of Zn^2+^, Zn(OH)_4_^2–^, Zn_4_(OH)_6_SO_4_·4H_2_O, and Zn(OH)_2_, which contain a higher concentration of the charged or neutral particles that are not dissolved into the bulk electrolyte (shows darker color). They are migrated along the direction of the electrical field. The following chemical reactions may occur simultaneously during the plating period in the ZnSO_4_ electrolyte, according to^30−32^
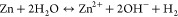
1
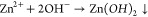
2
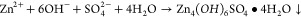
3

It is well recognized that X-ray diffraction (XRD) could help improve the understanding of chemical and crystallographic information. For instance, Shi et al.^33^ suggested that the deposited matter on different metal current collectors is all indexed to Zn metal. Hao et al.^34^ illustrated that on top of plated Zn, there is a thin layer of Zn_4_SO_4_(OH)_6_·3H_2_O formed, depending on the soaking time in the electrolyte. Chaba et al.^35^ found that an XRD pattern exists for Zn metal under a current density of 0.1 A cm-2 in the presence of 1 M ZnSO4. These studies have similar conditions to our work in terms of current density and electrolyte concentration. Therefore, we assume that the composition in this study is similar (or the same) as in previous studies. In other words, there will be the same pattern of Zn metal deposition as the Zn substrate deployed. As these clusters/particles continue to deposit unevenly on the primary Zn dendrite, the left branch grows quickly toward the counter electrode side until the cell short circuit at *t* = 25 min. It is worth noting that the diffusion and aggregation of charged clusters prefer nonlinear migration routes, as shown in Figure 1e–i. The divergent electric field leads to the formation of the low-dense (“sponge-like” structure) and the high-dense (compact structure) Zn morphologies on the left and right branches, respectively.

Using the same setup, Figure 2 and Movie 2 demonstrate another scenario of Zn electrodeposition with current densities increasing from 0.03 to 0.18 mA cm^–2^ at *t* = 220 min. At a low current density of 0.03 mA cm^–2^, there is almost no microstructural change within 30 min (Figure 2a,b). Under this current, the charged clusters transportation is expected to be weaker than CASE 1, so nucleation and subsequent crystallization are restricted. After 60 min, a much thinner layer of Zn is electrochemically plated onto the lower triangle substrate (Figure 2d). Interestingly, in CASE 2, there are some hydrogen bubbles observed next to the counter Zn electrode. It is well known that Reaction 1 occurs on the zinc anode^36^ and the hydrogen evolution is strongly correlated with the pH change at the surface of the Zn electrode.^37^ Therefore, we suspect there is a minor pH change in the aqueous electrolyte environment. The formation pathway of Zn dendrites indicates a diffusion-controlled growth mode; the charged clusters slowly accumulate on the Zn triangle substrate, forming “coral-like” structures with multiple dendritic tips (Figure 2e,f) until *t* = 120 min. When the current is deliberately increased to a higher value (0.18 mA cm^–2^), the “ring” appears near the liquid–solid interfacial area at *t* = 220 min (Figure 2g). Like CASE 1, Zn tip branching and splitting (Figure 2h–l) occur before cell short-circuiting due to ion transportation. The charged clusters preferentially plate onto the closest Zn substrate with the larger curvature,^38^ as confirmed by X-ray tomography results (Section 3.2).

Figure 3 and Movie 3 show the formation and dissolution of Zn electrodeposits under the highest current density of 0.3 mA cm^–2^ (CASE 3). To distinguish CASE 3 from the previous two CASEs, no color was assigned to the images extracted from a video. In Figure 3b, after the “ring” appears at *t* = 10 s, the electrodeposition process was immediately accelerated due to the uneven charged clusters distribution. The speed of dendritic growth is much faster than in CASE 1 and CASE 2. Although the initial distance between two Zn electrodes is almost identical (ca. 3.5 mm), the time to short-circuit for CASE 3 (ca. 120 s) is an order of magnitude shorter than CASE 1 (ca. 1500 s), and dendritic splitting and branching are more frequent than in CASE 1 and 2. Before the Zn dendrite approaches the opposite side, a negative current of the same magnitude (−0.3 mA cm^–2^) is applied to the system. The partial dissolution of Zn can be observed in Figure 3j–l, demonstrating that the stripping of Zn is not fully irreversible. The primary dendritic networks cannot dissolve completely after 120 s of stripping (Figure 3k), resulting in the formation of “dead” metal particles/dendrites, which is in accordance with previous work^9^ and further confirms the inherent accumulation effect.^19^ The “dead” Zn at a particular time could be quantified (image processing shown in Figure S3), with the area fraction of “dead” Zn accounting for 3% at *t* = 120 s. This result could act as a baseline for future comparisons when an advanced coating or new electrolyte will be used. It has been reported that the “dead” Zn particles could lower the Coulombic efficiency (CE), leading to degradation of battery performance.^39^ However, CE is ca. 100% for the Zn||Zn symmetric cells due to the excess Zn from both Zn electrodes.

In Figures 1–3, the deposited Zn exhibits the inhomogeneous dendritic growth, with the preferred branch having a larger size than the other branches. This might be attributed to the inhomogeneous concentration of electrolyte after stripping.^40^ The applied potential induces ion migration, which alters the electrolyte concentration and leads to the preferred growth.^41^ The non-uniform ion flux or large polarization has led to uneven Zn deposition and fast dendrite propagation (as presented in CASE 1–3). Therefore, it is essential to regulate charged ion transportation to achieve smooth and compact Zn deposition. There are some strategies to stabilize the inhomogeneous Zn deposition during plating; for example, developing the anode-free Zn battery may restrict the harmful dendrite growth and side reactions by limiting the excess of Zn metal.^42^ Also, replacing the electrolyte^24^ and applying an external magnetic field^43,44^ may help suppress the uneven Zn deposition and thus enable more stable cycling. For instance, the application of Zn(OTf)_2_ electrolytes in Zn batteries shows that Zn platelets nucleate more homogeneously and grow smaller than those in ZnSO_4_ electrolytes.^45^ Nevertheless, the understanding of these engineered methods to stabilize Zn on the anode side can be improved by adopting our current imaging technique.

**Figure 4:**
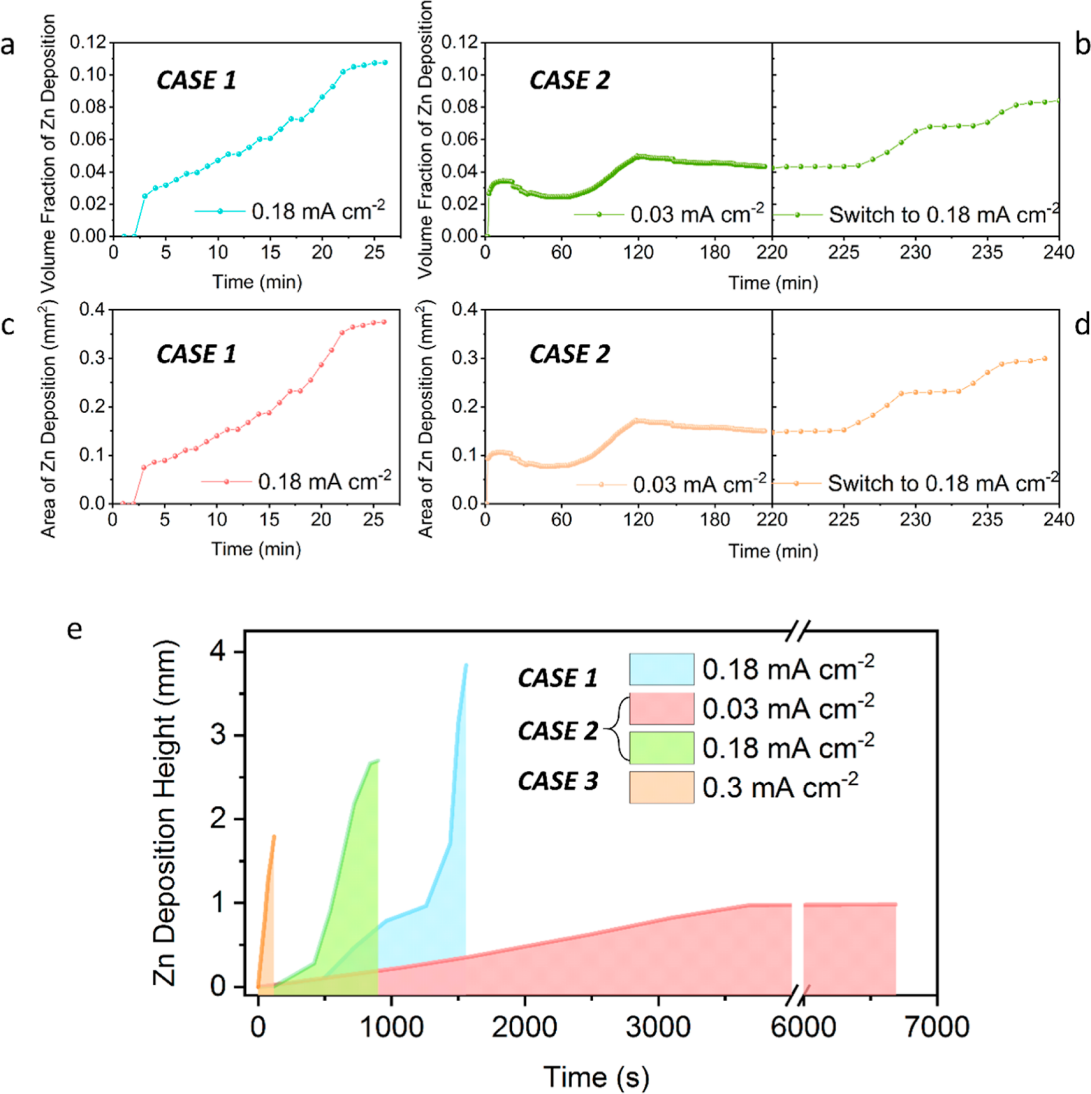
Corresponding quantification of operando imaging datasets. Quantification of newly formed zinc deposition on the original zinc tip, both the (a,b) volume fraction (*V*_2D–f_) and (c,d) area (*S*_2D-Area_) of zinc depositions were plotted as a function of plating time. (e) Associated dendrite height against their plating time under various current conditions. Note: the highest Zn point in each figure was selected for measurements.

Figure 4 presents the automated image quantification for the above three CASEs, including the 2D Zn deposition area (*S*_2D-Area_), the 2D volume fraction (*V*_2D–f_), and the Zn dendritic height (*h*). It is worth noting that *V*_2D–f_ is based on the pixel representation for features without a unit, which shows the percentage of Zn deposits that account for the entire FOV domain. While the *S*_2D-Area_ presents the area of deposited Zn with a unit (mm^2^), despite *S*_2D-Area_ and *V*_2D-f_ have an identical trend in their profiles.

In CASE 1 (0.18 mA cm^–2^), in Figure 4a–c show a steep increase after 2 min, indicating the prior observation by the appearance of a “ring” at *t* = 2 min. It suggests that this temporal point is the critical moment for dendritic growth. Afterward, the Zn dendrite gradually grew via diffusion until reaching the growth plateau at 23 min before short-circuiting. In CASE 2 (Figure 4b,d), under a current density of 0.03 mA cm^–2^, the Zn deposition evolved slowly until reaching the first growth plateau at *t* = 125 min. From 125 to 225 min, there are almost no changes, indicating the limited migrating-deposition is diffusing at a very low speed. Once it is increased to a higher current density of 0.18 mA cm^–2^, the Zn deposition resumes until *t* = 240 min (second plateau). Here, it can be noticed that CASE 2 shares smaller values (both *S*_Area_ and *V*_f_) compared to CASE 1. Therefore, variable current might help mitigate the abrupt dendritic growth (as seen in CASE 1). Figure 4e shows the heights of Zn dendrites for each CASEs over their plating time, with the slopes of the curves representing the growth velocities for each CASE. The highest growth velocity is seen in CASE 3 (0.3 mA cm^–2^). It is also noted that CASEs 1 and 2 have similar shapes under the same current; they both have a critical moment for dendritic growth before the sharp increase in deposition corresponding to the appearance of the “ring” feature in their microstructure.

### 3.2 4D Observation of Zn Electrodeposition

Operando digital microscopy (Section 3.1) provides dynamic information in 2D, thus morphological changes perpendicular to the camera direction cannot be obtained. Also, the compact setup may constrain free Zn growth, which should be considered. To overcome these limitations, X-ray tomographic imaging was employed to study Zn electrodeposition.

**Figure 5:**
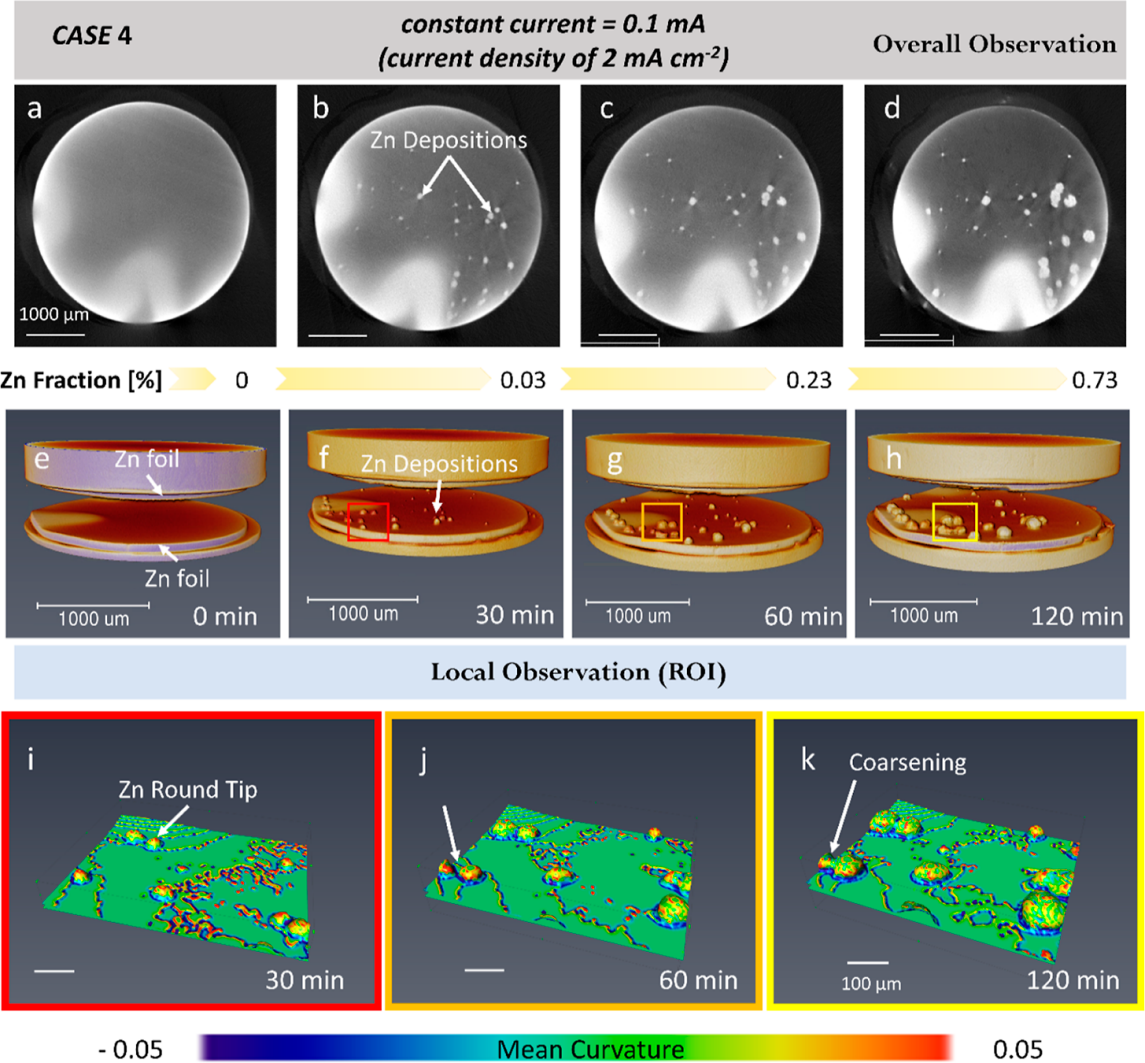
4D X-ray tomographic imaging of the slow zinc depositions under a current of 0.1 mA (with a current density of 2 mA cm^–2^). A symmetric Zn||separator||Zn Swagelok cell was used for the CT experiment. (a–d) 2D cross-sectional image and (e–f) 3D rendering image sequence showing the morphological evolution after slow Zn electrodeposition for 0, 30, 60, and 120 min in the entire FOV. The plated Zn particles on the Zn electrode are observable. (i–k) Selection of region of interest (ROI) demonstrating the distribution of mean curvature (from −0.05 to 0.05) of the Zn dendrite as a function of time at 30, 60, and 120 min. The scale bars represent 1000 and 100 μm for (a–d) and (i–k), respectively.

Figure 5 and Movie 4 demonstrate the Zn electroplating at very low current density (2 mA cm^–2^) within the pristine Zn||separator||Zn cell. The electric field is between the top and bottom plates. The “particle-like” features (brighter color) are the new deposits and their resultant X-ray attenuation. In Figure 5, the image sequences of 2D (first row) and 3D (second row) show the overall microstructure of Zn electrodeposits, while the third row illustrates the local 3D observations which are extracted from the entire domain. The Zn deposits have a round morphology, and no sharp dendrites are identified, as shown in Figure 5e–h. CASE 4 shows a lower volume fraction of deposited Zn is expected when using a lower current density (2 mA cm^–2^), indicating its influence on the Zn dendritic morphology. For the first time, we employ the mean curvature to characterize and quantify the electroinduced changes. Mean curvature is defined as: (), where  and  are the two principal curvatures at any point on the dendrite.^46^ The red color represents large positive values (*H*), and the green color indicates neutral values, while the blue color indicates large negative values (*H*). The normalized local curvatures are applied to the microstructure in CASE 4. The concept of curvature has been widely used in the materials solidification community to quantify dendritic growth and its coarsening. As it provides direct value and visualization for dendritic shape. The application of mean curvature in dendritic microstructure is common in the materials science community, and the mean curvature has been used for electrochemical engineering.

**Figure 6:**
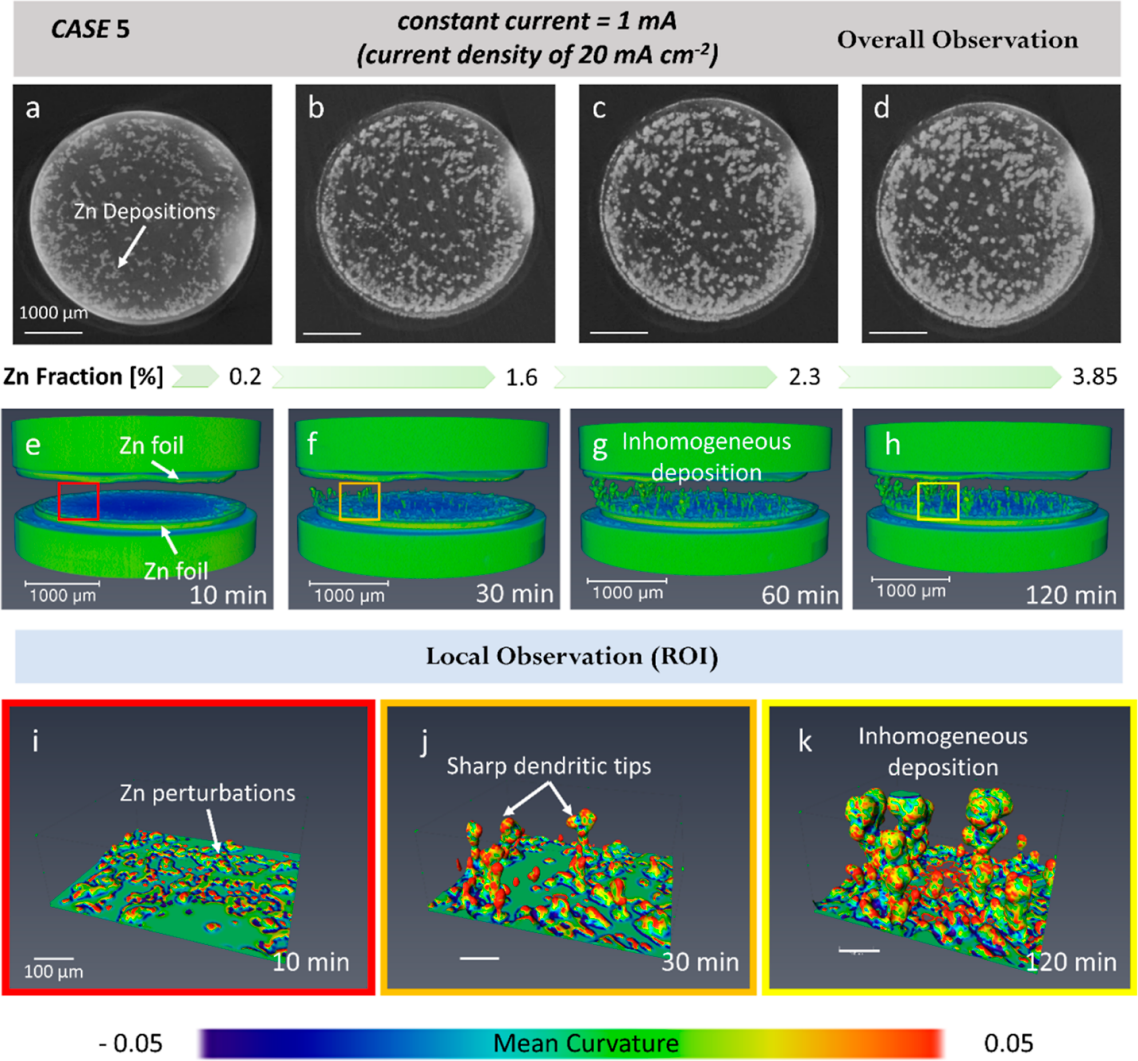
4D X-ray tomographic imaging of the fast zinc depositions under a current of 1 mA (with a current density of 20 mA cm^–2^). A symmetric Zn||separator||Zn cell with a glass-fiber separator in the middle was inserted in a Swagelok cell holder. (a–d) 2D slices and (e–f) a 3D rendering image sequence showing the morphological evolution after Zn electrodeposition for 10, 30, 60, and 120 min in the entire FOV. The plated Zn dendrites on the Zn electrode are observable. (i–k) Selection of region of interest (ROI) demonstrating the distribution of the mean curvature of the Zn dendrite as a function of time at 10, 30, and 120 min. The scale bars for (a–d) and (i–k) are 1000 and 100 μm, respectively.

Figure 6 and Movie5 illustrate Zn electrodeposition at a current density of 20 mA cm^–2^ in a Zn||separator||Zn cell. The first row of Figure 6 shows the cross-sectional slice extracted from the middle of the field of view (FOV). Like CASE 4, Zn protrusions in CASE 5 preferred to deposit at the edge of the sample rather than in the middle region after 10 min (Figure 6a,e). The diameter of Zn depositions gradually increases with the plating time, and the deposited Zn becomes coarser and aggregated after 60 min (Figure 6c). Correspondingly, the second row of Figure 6 shows the 3D microstructures of Zn deposits. The “droplet-like” dendritic morphologies are similar to those observed in previous synchrotron studies.^23^ At *t* = 10 min, in Figure 6e, the charged clusters transition might be under a quasi-equilibrium condition since protrusions with similar heights appear at the edge of the sample. After 30 min (Figure 6f), the Zn dendrites on the left side are higher than those on the right side, which may be caused by the non-uniform flatness of the Zn counter electrode, resulting in an uneven distribution of the electric field. Moreover, a higher growth rate could generate hyper-branches via tip splitting (Figure 6g–h). This phenomenon of heterogeneous growth has been observed in previous Figures 1–3. In the third row of Figure 6, three regions of interest (ROI) are selected from the associated FOV against the plating time to reveal the local microstructure evolution. The renderings show that in Figure 6j–k the Zn dendrite arm with the finest tip radii has the highest positive value (*H*), while the dendritic roots have the largest negative *H*. Comparing Figures 5 to 6, we can conclude that the dendritic surface exhibits a larger *H* in the local region when a higher current (CASE 5) is applied. In other words, a lower current (CASE 4) induces a flatter tip surface, which could be less detrimental to separator rupture. The dendritic zinc deposits exhibit an elastic modulus of up to 105 GPa^39^—a considerable value, higher than other metal deposits (such as 6.8 GPa of lithium^47^ and 30 GPa of magnesium^48^)—indicating that Zn deposits have a higher possibility to puncture commonly used polymeric separators than other metal batteries if the deposits are not well controlled.

**Figure 7:**
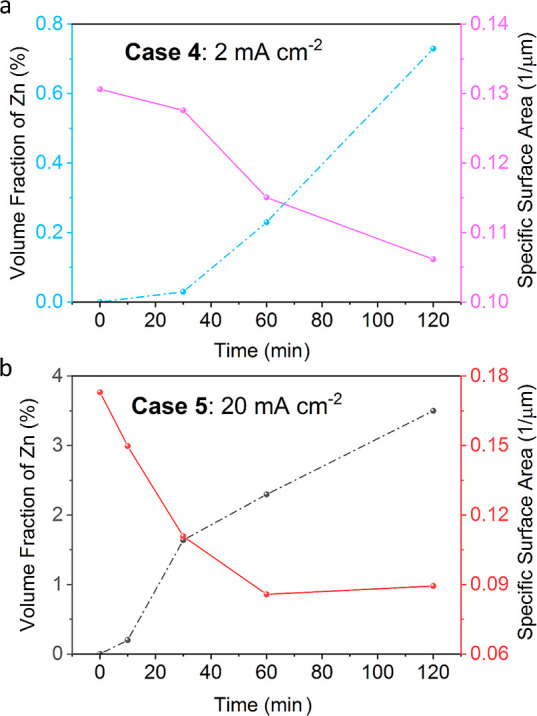
Corresponding quantification of *time-lapse* CT imaging datasets under 2 and 20 mA cm^–2^ (constant currents of 0.1 and 1 mA) including the Zn volume fraction (*V*_3D–f_) and specific surface area (*S*_3D-spec_).

As expected, Figure 7 shows the current has a strong influence on the 3D volume fraction (*V*_3D–f_) and specific surface area (*S*_3D-spec_) of the Zn electrodeposits. The higher the current that is applied, the larger value of *V*_3D–f_ is obtained. For instance, the *V*_3D–f_ in CASE 5 is almost five times larger than that in CASE 4 under the same plating time. The *V*_3D–f_ continually increases as the plating time, while the *S*_3D-spec_ has the opposite trend. The decrease of *S*_3D-spec_ is mainly attributed to the dendrites merging and self-coarsening. It should be noted that the fractions of Zn deposits characterized by optical microscopy (*V*_2D–f_) and tomography (*V*_3D–f_) are 4.8 and 3.85% under the same current (1 mA) and platting time (120 min), respectively. The value by CT is slightly smaller than that by optical microscopy could be attributed to the resolution, as some tiny deposits may not be resolved by our micro-CT. Nevertheless, the comparable results not only suggest the volume fraction of Zn deposits is independent of the measuring methods and cell configurations but also confirm the reliability and credibility of our measurements.

### 3.3 Correlative Electrochemical Cycling and Imaging

**Figure 8:**
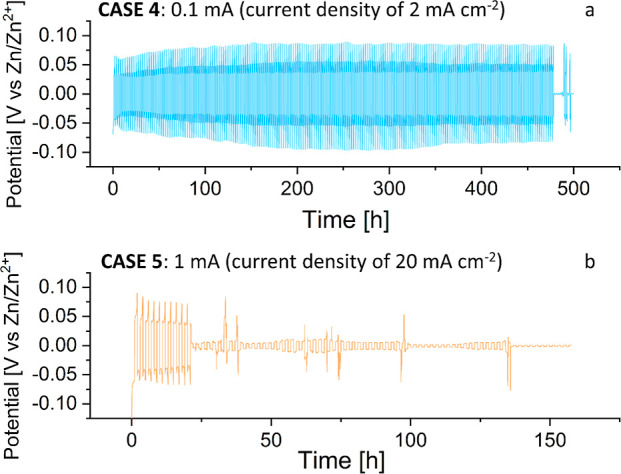
Long-term Zn plating/stripping behavior using the constant currents of (a) 0.1 and (b) 1 mA (current densities of 2 and 20 mA cm^–2^). The two cycled cells are corresponding to CASE 4 and CASE 5 with identical chemical composition, cell configuration, and applied potentials. The voltage–time profiles of electrodeposition and stripping of Zn on Zn foil in a 3 M ZnSO_4_·H_2_O electrolyte.

In Figure 8, long-term Zn plate/strip cycling in the Zn||separator||Zn Swagelok cell is presented using two representative current densities (2 and 20 mA/cm^2^). Under a low current density of 2 mA/cm^2^, short-circuiting occurred after 480 h of operation, while applying a high current density of 20 mA/cm^2^, the cell underwent short-circuiting after 25 h. Our results are comparable to previous studies, although different cell configurations were used. Zhao et al.^49^ demonstrated that the Zn||Zn cell could enable cycling for 300 and 25 h when applying the current densities of 0.885 and 20 mA/cm^2^ to the coin cells. Wang et al.^50^ found a symmetric Zn//Zn cell has excellent cycling performance without overpotential increase over 400 h using the 0.5 mA/cm^2^. In this study, although the Swagelok-type cell, basic electrolyte, and Zn electrodes without advanced coating technology were used, stable electrochemical performance was achieved (Figure 8), with good Zn reversibility without a severe increase of overpotential over time. The increase of overpotential (Figure 8a) in each plate or strip suggests irreversible reactions, causing Zn loss (“dead” Zn) and increased resistance.

Since the chemistry, cell configuration, and cycling conditions are identical (same as CASEs 4 and 5), it will be robust to correlate the cell performance (Figure 8) with the 4D imaging of microstructures (Figures 5 and 6). The high current density (20 mA cm^–2^) could generate sharp dendrite tips with a high positive curvature, which will continually grow along the current direction as they cannot fully dissolute after stripping (supported by CASE 3). Additionally, the growth speed of dendrites at a higher current is much faster than that at a lower current CASE (supported by Figure 4e), and the sharper dendrites (Figure 6k) have a higher probability of penetrating the separator than those of rounder Zn deposits (Figure 5k). Furthermore, the electric field will be localized at the sharp tip of the Zn dendrite (where the electric field intensity is much greater than in the adjacent regions),^20^ which facilitates the electrodeposition and leads to further dendritic growth and worsens the already inhomogeneous deposition. There will be a more uniform distribution of the electric field for rounder Zn dendrites. Hence, according to the aforementioned reasons, it is understandable to see short-circuiting occur much earlier in a higher current condition.

## 4 Conclusions

Microscale *operando* optical microscopy and in situ X-ray tomography studies have provided deeper insight into the formation and dissolution of dendritic Zn in alkaline solutions during galvanostatic plating/stripping. By applying automated quantification to the resulting image data, we found that Zn electrodeposition at the early stage is mainly attributed to activation, while the subsequent dendrite growth is dominated by diffusion. The effect of current densities on Zn deposition in a symmetrical Zn cell was studied; the higher current density leads to a shorter initiation time and larger Zn dendrites. Additionally, a high current facilitates the formation of sharp dendrites with a larger mean curvature at their tips and leads to dendritic tip splitting and a hyper-branching morphology. The dynamic information is captured, including the heterogeneous mass transportation and the creation of “dead” Zn particles through partial dissolution. This study strengthens the current understanding of metal-battery failure mechanisms, providing valuable experimental datasets for use in modeling activities, such as phase field and imaging-based simulations, and offers a feasible, low-cost approach to characterize other multivalent metal-ion batteries with a metal anode in real-time in the laboratory. It is envisaged that this combination approach will have a broader application and impact on the development of new battery materials. For instance, it is possible to observe how the new electrolyte or the novel interface coating could mitigate dendritic growth.

## Supporting Information Available

Animation links for both the operando and time-lapse experiments, electrochemical profiles for the operando microscopy experiments, photographs of experimental setups, and the method to quantify the “dead” zinc (PDF)
