# Supplementary material for: Observation of Zn Dendrite Growth via Operando Digital Microscopy and Time-Lapse Tomography
Source: ACS Appl Mater Interfaces. 2023 Mar 9;15(11):14196–205. doi: 10.1021/acsami.2c19895 (PMC10037236; doi:10.1021/acsami.2c19895)
Supplement: Supplementary file 1 — am2c19895_si_001.pdf [file am2c19895_si_001.pdf]

Supporting Information

Observation of Zn Dendrite Growth *via* Operando Digital Microscopy and Time-Lapse Tomography

Wenjia Du[1, 2], Zhenyu Zhang[1, 2], Francesco Iacoviello[1], Shangwei Zhou[1], Rhodri E. Owen[1, 2], Rhodri Jervis[1, 2], Dan J. L. Brett[1, 2], Paul R. Shearing[1, 2*]

1. Electrochemical Innovation Lab, Department of Chemical Engineering, University College London, London, WC1E 7JE, U.K.
2. The Faraday Institution, Quad One, Harwell Science and Innovation Campus, Didcot, OX11 0RA, U.K.

Key words: Zinc electrodeposition; Dendrites; Plating; Stripping; X-ray computed tomography

Corresponding Authors: Paul R. Shearing (p.shearing@ucl.ac.uk)

Supporting Movies

Supporting Movie1: https://figshare.com/s/4604ddd6118f4323f58e

Supporting Movie2: https://figshare.com/s/9ae59adce7f770919d4c

Supporting Movie3: https://figshare.com/s/e428d1f37a71cbef7ca9

Supporting Movie4: https://figshare.com/s/31114cd2b2822c4f4baf

Supporting Movie5: https://figshare.com/s/7882bd557219f9da6431

Supporting Figures

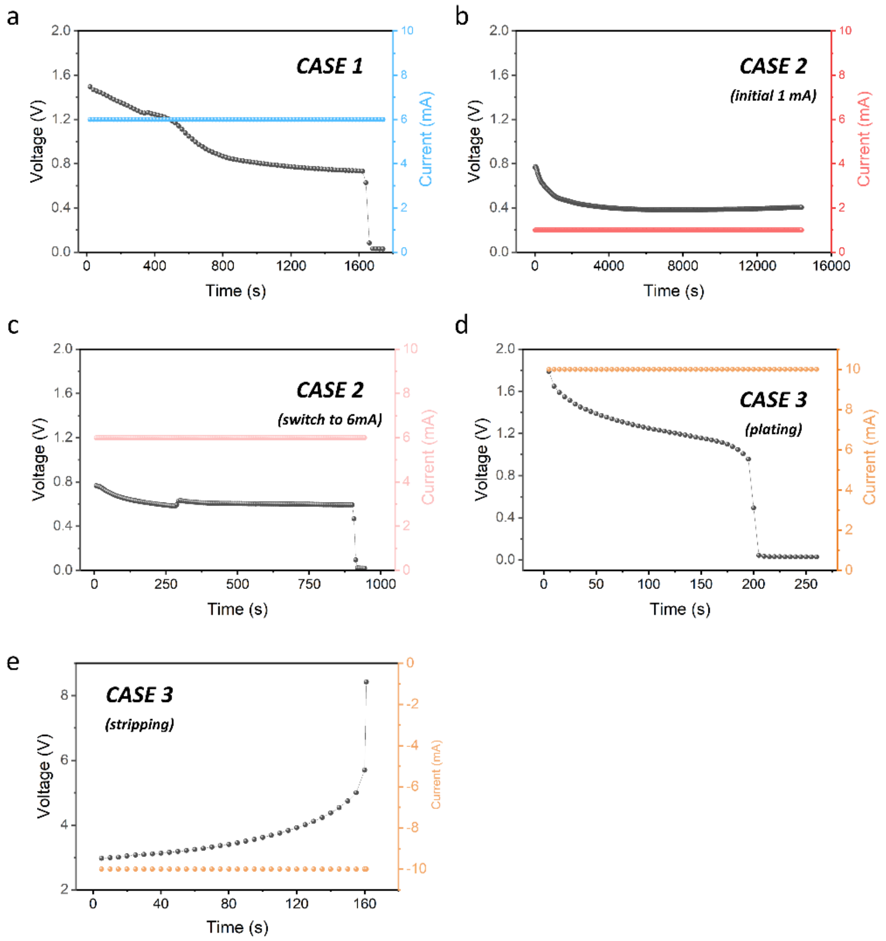

Figure S1. The Zn plating profiles during the electrodeposition in the Zn||Zn cell under the constant current of (a) 6, (b) 1, (c) 6, (d) 10, and (e) -10 mA. The corresponding current densities are 0.18, 0.03, 0.18, 0.3 and -0.3 mA cm^{-2}.

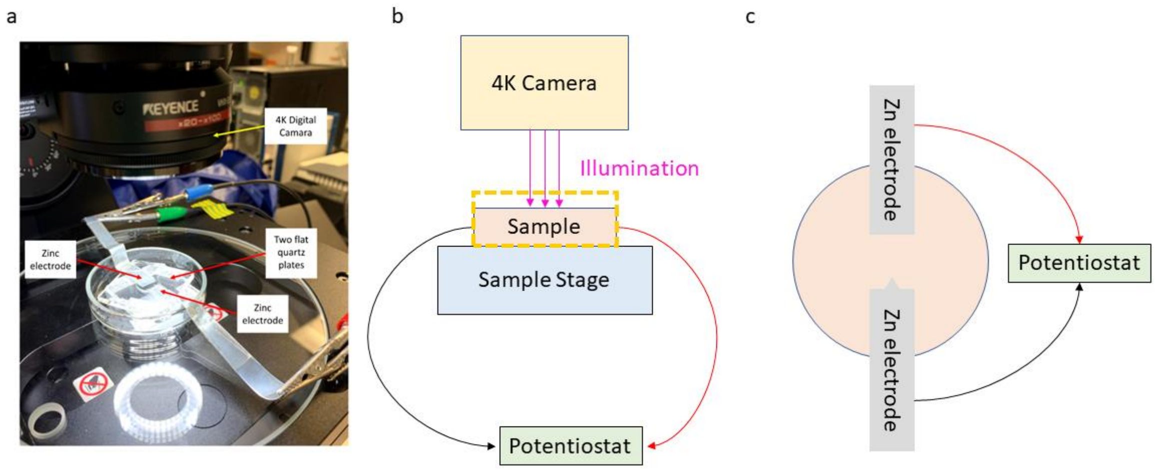

Figure S2. (a) The genuine experimental setup of digital microscopy for *operando* zinc electro-deposition and dissolution in symmetric Zn||Zn cell. For demonstration purposes the distance between the sample and the lens is increased. (b) Schematic of the experimental setup. (c) Schematic of the sample region (from the top view), the electrolyte covers the region of entire circle.

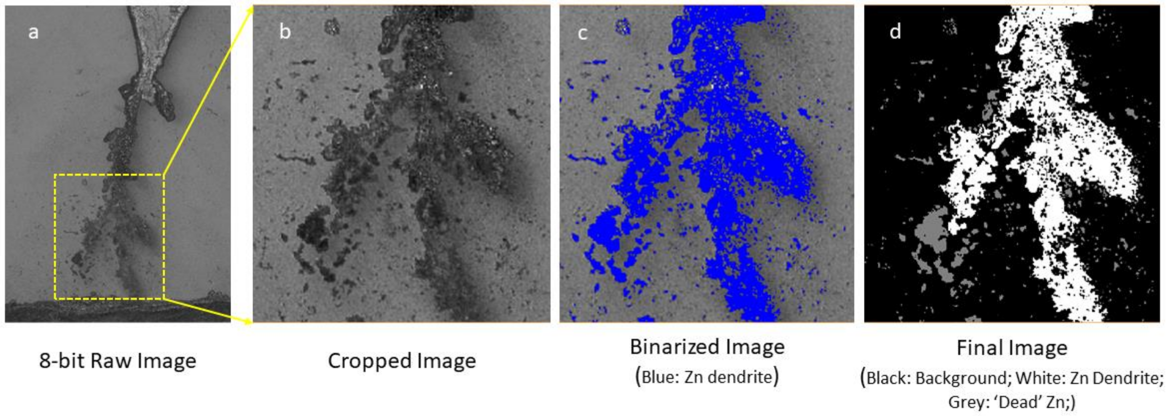

Figure S3. The imaging processing to quantify the fraction of dead Zn. (a) 8-bit raw image from microscopy (the dashed line highlights the crop region). (b) Cropped image from (a). (c) The binarised image segmentation to classify all Zn features. (d) The image contains the dead Zn and main Zn dendrites at t = 120 s. The fractions of dead Zn and dendrite Zn are 3 % and 15 %, respectively.

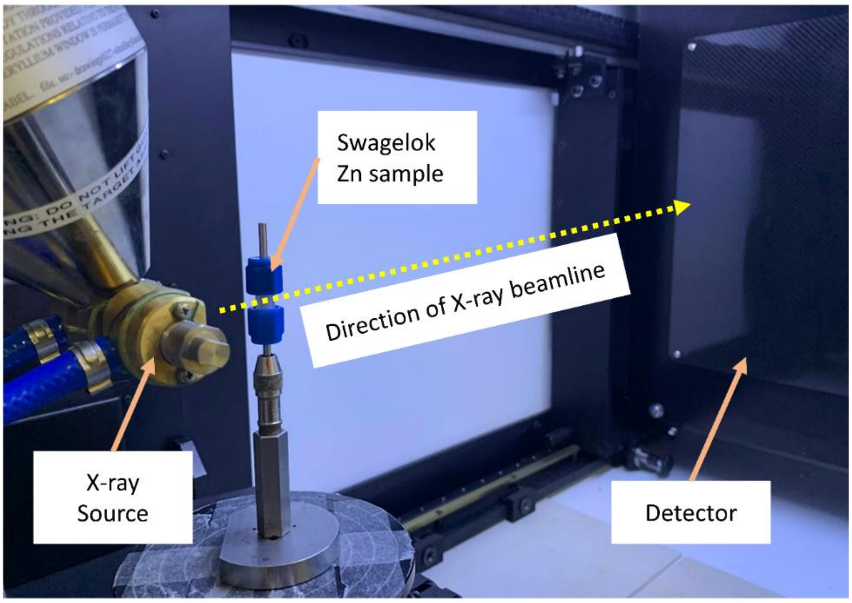

Figure S4. The experimental setup of lab-based X-ray tomography for the Swagelok sample (Zn||separator||Zn cell) by using the Nikon XT H 225 instrument.
